# An Aluminum Microfluidic Chip Fabrication Using a Convenient Micromilling Process for Fluorescent Poly(dl-lactide-*co*-glycolide) Microparticle Generation

**DOI:** 10.3390/s120201455

**Published:** 2012-02-01

**Authors:** Yung-Sheng Lin, Chih-Hui Yang, Chih-Yu Wang, Fang-Rong Chang, Keng-Shiang Huang, Wan-Chen Hsieh

**Affiliations:** 1 Department of Biological Science & Technology, I-Shou University, Kaohsiung 84001, Taiwan; E-Mails: linys@sunrise.hk.edu.tw (Y.-S.L.); chyang@isu.edu.tw (C.-H.Y.); wanjenxie@gmail.com (W.-C.H.); 2 Department of Applied Cosmetology and Master Program of Cosmetic Science, Hung-Kuang University, Taichung 43302, Taiwan; 3 Department of Biomedical Engineering, I-Shou University, Kaohsiung 82445, Taiwan; E-Mail: crab@isu.edu.tw; 4 Graduate Institute of Natural Products, College of Pharmacy, Kaohsiung Medical University, Kaohsiung 807, Taiwan; E-Mail: aaronfrc@kmu.edu.tw; 5 The School of Chinese Medicine for Post-Baccalaureate, I-Shou University, Kaohsiung 82445, Taiwan

**Keywords:** microfluidic emulsification, poly(lactic-*co*-glycolic acid) (PLGA), quantum dots (QDs), microsphere

## Abstract

This study presents the development of a robust aluminum-based microfluidic chip fabricated by conventional mechanical micromachining (computer numerical control-based micro-milling process). It applied the aluminum-based microfluidic chip to form poly(lactic-*co*-glycolic acid) (PLGA) microparticles encapsulating CdSe/ZnS quantum dots (QDs). A cross-flow design and flow-focusing system were employed to control the *oil-in-water* (*o/w*) emulsification to ensure the generation of uniformly-sized droplets. The size of the droplets could be tuned by adjusting the flow rates of the water and oil phases. The proposed microfluidic platform is easy to fabricate, set up, organize as well as program, and is valuable for further applications under harsh reaction conditions (high temperature and/or strong organic solvent systems). The proposed method has the advantages of actively controlling the droplet diameter, with a narrow size distribution, good sphericity, as well as being a simple process with a high throughput. In addition to the fluorescent PLGA microparticles in this study, this approach can also be applied to many applications in the pharmaceutical and biomedical area.

## 1. Introduction

Microfluidics has been increasingly gaining attention in crucial application areas ranging from chemical synthesis and analysis, medical diagnostics, environmental monitoring, to analytical microsystems for cell biology [1–4]. Over the past decade there has been a huge increase in the annual number of scientific publications on microfluidics [5]. Microfluidics is the science and the technology of systems manipulating nanoliters of fluids in channels with dimensions measured in tenths or even hundreds of micrometers [6]. With the maturation of the Micro-Electro-Mechanical System (MEMS) technology, microfluidics has become a new and different tool to offer precise control over the flows of fluids at very small scales [7] suitable for a wide range of applications [8–11].

The fabrication of microfluidic chips can be classified into direct substrate manufacturing and mold-based techniques [2,4,7,12]. Although mold-based techniques can be used for the mass production of chips, they often need costly mold masters for replication [4]. Lithography and laser ablation are the most typical direct substrate manufacturing techniques. Lithography has good ability to manufacture tiny micro-channels, but it usually involves many tedious processing steps, aggressive chemicals for the etching step, and it requires high tech facilities in a clean-room environment [4,13,14]. Laser ablation has the advantage of direct-write micromachining without a mask process and does not depend as much on the type of substrate material used [15]. However, microchannels fabricated by laser ablation have a greater surface roughness than those produced by the general mold-based techniques such as hot embossing, imprinting, or injection molding [13]. Computer numerical control (CNC)-based mechanical micromachining is another direct-write micromachining technique. Its advantages are similar to those of laser ablation, but with less surface roughness in the fabricated microchannels. Other advantages are its accessibility and rapid prototyping capability to quickly fabricate a diverse set of workpieces or substrates. Although mechanical micromachining is not very satisfactory for miniaturization work, it can be used for dimensional structures as small as 50 micrometers [5,16]. We believe that it is suitable for producing microfluidic droplets. To-date few studies have applied the conventional direct-write mechanical micromachining method for fabricating microchannels for producing microfluidic droplets.

Most microfluidic chips are made of silicon, quartz/glass, or polymer-based platforms [12]. Although these materials have their own unique characteristics, such as the good surface quality of silicone and quartz/glass, or the optical clarity of quartz/glass and polymer, there remain some inconveniences in their use. For example, quartz/glass or silicone-based products are usually expensive, have low impact strength, and require a long processing time [17]. Polymer-based products generally have low chemical resistance to organic solvents and may degrade and deform over time. Metal is an alternative candidate for the microfluidic chip substrate due to its excellent mechanical properties, reusability, and ease of fabrication in conventional mechanical micromachining. Metal is used especially in the harsh environment of high temperatures and where there is the need for good thermal conductivity [5]. There are some through-holes in metal-based plates for generating common *oil-in-water* droplets such as soybean, kerosene, and sunflower oil [18–20]. It is surprising that metal-based chips have not widely been proposed to produce microfluidic droplets.

Recently, we attempted to develop new research tools for biological and pharmaceutical applications by means of microfluidics [21–27]. In this paper, we describe an application of the micro-milling process and the microfluidic technique for generating fluorescent poly(lactic-*co*-glycolic acid) (PLGA) microparticles. The well-established flow-focusing fluidic junction was applied to create segmented droplets for monodispersed quantum dots (QDs)-loaded microparticles which are potentially used as micro-carriers for biological labeling and diagnostics. The proposed Al-based microfluidic chip is easy to fabricate, easy to set up, and is easily programmed to generate uniform microparticles in mass production. The fabricated Al microfluidic chips have the advantages of being durable and long-wearing, with a high chemical resistance to organic solvents, and a design that allows for easy disassembly, channel rinse and cleaning.

## 2. Experimental

### 2.1. Materials

Poly(vinyl alcohol) (PVA, 88%–89% hydrolyzed), PLGA, and hexamethyldisilathiane [S(Si(CH_3_)_3_)_2_] were obtained from Sigma (Sigma Chemical Co., St. Louis, MO, USA). Tri-*n*-octylphosphine oxide (TOPO, 98%), selenium powder (Se, 99.5%), and *n*-tetradencylphosphonic acid (TDPA, 98%) were purchased from Alfa Aesar (Alfa Aesar Inc., Ward Hill, MA, USA). Cadmium oxide (CdO, 99.5%) was obtained from ACROS (ACROS Organic Co., Morris Plains, NJ, USA). Dimethylzinc (Zn(CH_3_)_2_, 95%) and tributylphosphine (TBP, 98%) were purchased from STREM Chemicals (Newburyport, MA, USA) and SHOWA (SHOWA, Japan), respectively. All reagents were used as purchased, without any additional purification. For the preparation of TOPO-coated CdSe/ZnS QDs please refer to the Supplementary file.

### 2.2. Fabricating the Microfluidic Platform

**Figure 1.**
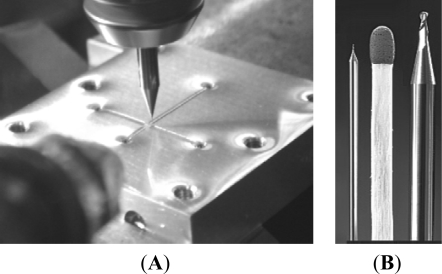
Fabrication of an aluminum-based microfluidic chip. (**A**) Photograph of the micro-milling process and (**B**) photographs of micro-cutters (two sides) and a scale bar (a match, center).

A CNC-based micro-milling method was used for the mechanical manufacture of the micro-sized fluidic platform on the Al substrate. Figure 1(A) is a photograph of the micro-milling process, and Figure 1(B) shows the 100 μm diameter micro-cutter (left) as well as the 1,000 μm diameter micro-cutter (right) (End mill cutter, DIXI Tool Co., Taiwan).

**Figure 2.**
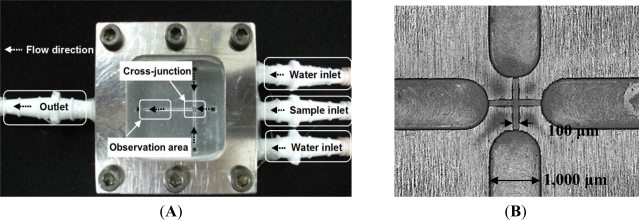

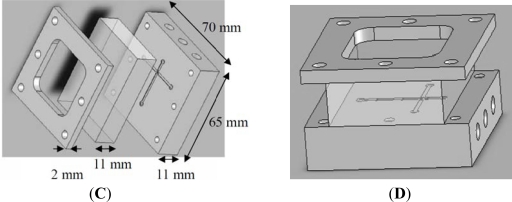
The proposed aluminum-based microfluidic device. (**A**) Photograph of assembled device; (**B**) close-up of the 100 μm wide cross-junction; (**C**) exploded view; and (**D**) perspective drawings of the proposed device.

We used a 4-axis machine tool (Ta Liang Technology Co., Ltd., Taiwan) to manufacture the microchannels on a surface-polished 6061 aluminum alloy substrate. A high-speed (up to 40,000 rpm) spindle was installed on the 4-axis machine tool to provide micro-milling cutters with a small run-out at high turning speeds for creating the microchannels by high-speed micro-milling. The cutting-depth was set to 5 μm per cut for the micro-cutting process. We programmed 10 passes on the CNC micro-milling machine to reach a 50 μm depth. After the surfaces of the microchannels were finished, four 1 mm inner channels were drilled in the broadening channels (1,000 μm in width) to slow down the flow and enhance observation at the downstream of the cross-junction. Figure 2(A,B) show photographs of the proposed Al-based microfluidic device with a 100 μm wide cross-junction. Figure 2(C,D) show the expanded and the perspective drawings of the device, respectively. The microfluidic chip consists of three layers, the cover layer (epoxy resin containing six screw orifices for binding), the middle layer (glass window, 10 mm thick) and the bottom layer (aluminum disk structure containing one outlet, three inlets, and six screw orifices). These three layers were then integrated using six M4 screws (0.5 mm pitch, 4.0 mm diameter, and tightened by a force of 1.0–1.2 Nm). Fluid interconnections between the chip and the tubes were made by straight connectors. Because the glass window and aluminum disk had been surfaced to get a perfect plane surface, this microfluidic chip could be leak proof. The microfluidic platform was easy to fabricate, easy to set up, as well as easy to organize and program.

### 2.3. Experimental Procedure

In the *o/w* emulsions, the oil phase (dispersed phase) was a mixture of PLGA (0.5 mg/mL) and CdSe/ZnS-TOPO QDs (0.5 mg/mL) in dichloromethane at a 10:1 ratio. The water phase (continuous phase) contained PVA (1% wt/v). Three independently controlled syringe pumps (Kd Scientific KDS230, New Hope, PA, USA) were used to simultaneously pump the flows of the two phases into the microfluidic chip. The dispersed phase and the continuous phase were injected into the middle channel and two side channels, respectively. The shear force created by the water flow gradually necks the drop at the cross-junction stretch, and then finally breaks into a droplet. The generation of droplets in the junction is a result of the competition of viscous stresses associated with the imposed flow and the capillary stresses due to the surface tension of the oil/water interface. These droplets were then dropped into the reservoir where they continued on hardening. After evaporating dichloromethane in a 50 mL beaker containing 30 mL solution at 37 °C for 24 h, the CdSe/ZnS-TOPO QDs-loaded PLGA microparticles were collected by centrifugation, washed three times with distilled water, and lyophilized for 24 h to complete dichloromethane removal for further optical examination [28].

## 3. Results and Discussion

**Figure 3.**
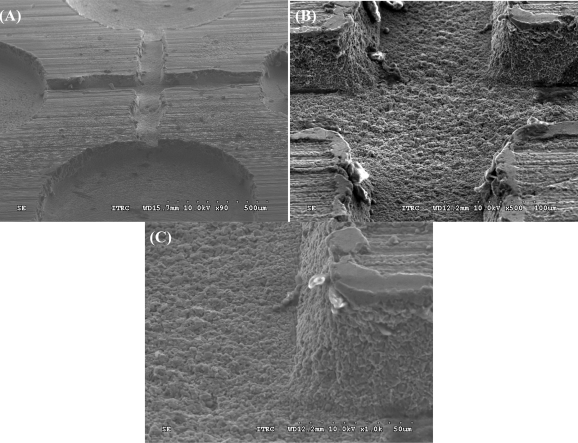
SEM photos of the aluminum microfluidic device. (**A**) Microchannels, (**B**) cross-junction, and (**C**) side channel wall.

Micro-milling techniques are widely used for micromachining. Although soft lithography can do the same work in mass production to reduce the unit product price, the capital cost for establishing the environment and equipment is high [4,13,14]. Nevertheless, micro-milling techniques have the advantage of easy accessibility and rapid prototyping capability in fabricating certain prototype workpieces [29]. For example, biochips with microfluidic channels can be directly machined by the micro end-milling process and powder blasting process [29]. It is therefore somewhat of a surprise that metal-based microfluidic chips have not widely been proposed for producing droplets. Here, we describe a new application of the micro-milling process for microfluidic droplets. This study used conventional CNC-based micro-milling technology to machine an Al-based microfluidic chip for application in the pharmaceutical/medical area. The properties of this chip have the advantages of both robustness and reusability. Figure 3(A) shows the fabricated 3D microchannels in the Al chip. The size of the cross-junction microchannels in this chip measures 100 μm in width and 50 μm in height (Figure 3(B)), and the side channel wall is close to 90° vertical (Figure 3(C)). The roughness Sq, defined as the quadratic mean of the deviation from the mean in the EUR 15178 EN report, is 2.14 μm measured by a TalyScan 150 profilometer. These results show that the fabricated micro-sized platform of the Al microfluidic chip is sufficient to generate micro-droplets by breaking up immiscible liquids in order to produce fluorescent monodispersed microparticles. The roughness of the channel will cause a certain disturbance to the flow-streams. Our experiment showed that when the size of the droplet dropped below 300 μm in diameter, the variation coefficient of the droplet distribution was more than 3%. After shrinking, the variation coefficient of the obtained microparticles was more than 10%.

The generation of uniform PLGA microparticles was based on the use of a flow-focusing system, which is one of the most frequently used microfluidic strategies for producing immiscible fluid segments, by exerting control on the *o/w* emulsions to generate orderly droplets. The mechanism of the droplet formation in a liquid/liquid emulsification system has already been studied extensively from both theoretical and experimental perspectives [30,31] due to the wide variety of engineering applications.

**Figure 4.**
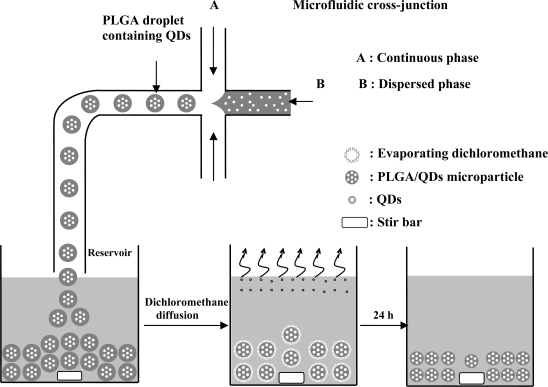
Schematic drawing of droplet formation in a microchannel cross-junction. Based on the principle of microfluidics for exerting control over the shear focusing force, a large set of uniform self-assembling microspheres can be obtained. The PLGA microparticles are formed after removal of the dichloromethane through diffusion and evaporation.

As shown in Figure 4, the stable and symmetric shear force of the water flow on the oil flow in the cross-junction brings about a steady formation of uniform self-assembling droplets, or so-called micro-emulsions. The size and the gap of these droplets are tunable by varying the flow rates of the two liquid phases. The PVA surfactants in the aqueous solution can stabilize the PLGA droplets and prevent unwanted coalescence between the droplets by reducing the interfacial tension between the oil and water phases [32]. When these droplets are transported to a reservoir solution through a Teflon tube, they precipitate spontaneously to the bottom of the reservoir since their density is higher than that of the solution. After they have undergone a complete solvent evaporation, the CdSe/ZnS-TOPO QDs-loaded PLGA microparticles can be observed under optical microscope and SEM.

**Figure 5.**
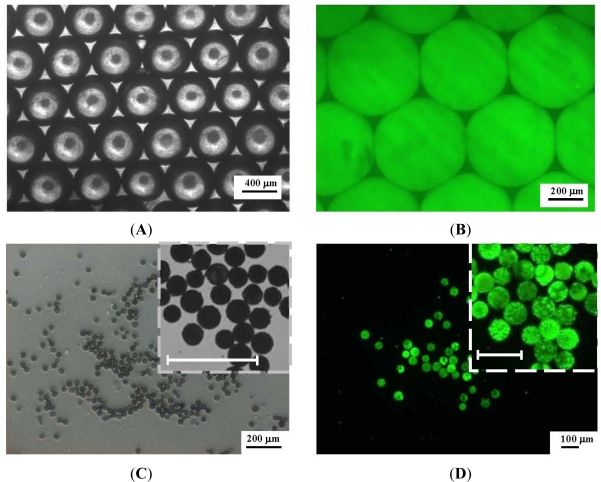
Micrographs of monodispersed PLGA droplets (**A**) bright-field (Scale bar = 200 μm) (**B**) fluorescent images (Scale bar = 200 μm), and microparticles (**C**) bright-field (Scale bar = 200 μm) and (**D**) fluorescent images (Scale bar = 100 μm).

Figure 5 shows the microscope images of the CdSe/ZnS-TOPO QDs-loaded PLGA droplets (Figure 5(A,B)) and the PLGA microparticles (after removal of the dichloromethane though diffusion and evaporation) (Figure 5(C,D)) collected in the reservoir under the conditions of a 0.08 mL/min oil flow rate and a 0.10 mL/min water flow rate. In the bright-field microscope images (Figure 5(A)), the droplets form a closely packed hexagonal structure. After exciting with the third harmonic, 350 nm, of a Surelite I-10 Nd:YAG laser, the imaging (Figure 5(B)) shows that the fluorescent signals of each PLGA droplet are consistent in both morphology and size, and exhibit excellent size uniformity. The average diameter of the PLGA droplets is 520 µm. During the evaporation procedure, the droplets shrink gradually and in the end form smaller and denser matrices (Figure 5(C,D)). The average diameter of the PLGA microparticles is 42.8 μm ± 2.3 µm. The reduction from 520 µm to 42.8 μm diameter, means a 91.8% shrinkage in diameter from droplet to microparticle. With the shrinkage as high as 90%, the particle properties are mainly controlled by the drying process rather than the droplet formation [28,33,34].

**Figure 6.**
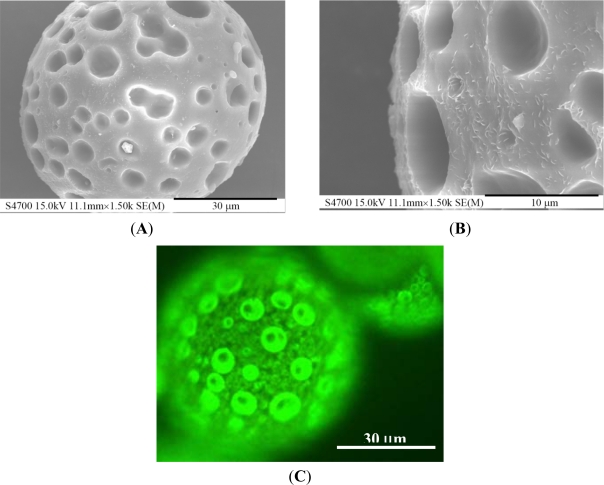
Microscope images of PLGA microparticles. (**A**) and (**B**) are SEM images. (**C**) is fluorescent image. The size of the sphere is 60 μm.

When examined under the scanning electron microscope, PLGA microparticles are not dense in structure but have an open porous surface (Figure 6(A)) as a result of the rapid dichloromethane evaporation in the liquid/liquid phase separation of the polymer solution. The maximum pore size on the surface reaches almost 10 μm (Figure 6(B)). The fluorescent image (Figure 6(C)) shows that a large number of QDs are distributed over the surface of the PLGA microsphere. Due to dragging by the dichloromethane during its evaporation, numerous QDs dispersed on the surface of the PLGA microparticles, and a certain portion of the QDs was entrapped in the interior matrix. The ODs loading percentage could be determined by dissolving the PLGA microparticles and measuring the fluorescence of the QDs [35]. The overall encapsulation of the QDs was 8.1% (weight percent) of the PLGA microparticles, which is close to the initial concentration of QDs in the oil phase solution excluding dichloromethane.

**Figure 7.**
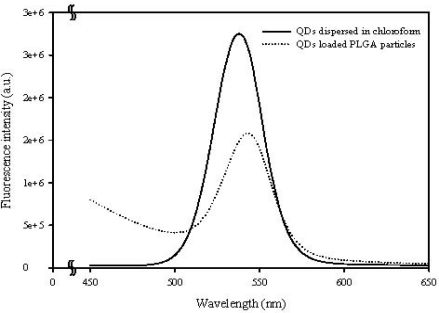
PL spectra obtained from as-prepared CdSe/ZnS-TOPO QDs (solid line) and CdSe/ZnS-TOPO QDs-loaded PLGA microparticles (dotted line).

Figure 7 compares the extinction spectrum of CdSe/ZnS-TOPO QDs before and after being entrapped in the PLGA microparticles. The maximum emission of the QDs has a slight red-shift from 542 nm to 549 nm due to the PLGA matrix. This shows that the characteristics of the QDs were not altered in the incorporation process, and that the QDs-loaded PLGA microparticles can provide a highly amplified signal for imaging applications, as shown in Figures 5(D) and 6(C).

Table 1 shows the relationships between the droplet/microparticle size and water/oil flow rates in this Al-based microfluidic chip system. When the oil flow rate is fixed between 0.06 mL/min and 0.08 mL/min, the size of PLGA droplets decreases as the water flow rate increases. This result is due to the fact that the larger water flow rate strengthens the shear force and accelerates the detachment of the droplets from the oil flow at the cross-junction. An increase in the water flow rate decreases the droplet formation time of the oil flow, and therefore the size of the PLGA droplets is smaller in a higher water flow rate [32]. The effect of a continuous flow rate on the droplet size is more significant at a higher PLGA concentration due to the increase in the viscosity of the dispersed phase. In addition, the size of the PLGA droplets increases with the oil flow rate for the given fixed water flow rate between 0.08 and 0.12 mL/min. Similar to the results of previous studies [21–27], the size of the droplets is greatly affected by both the water and the oil flow rates. Under the same droplet formation mechanism, at a low droplet formation rate in a quiescent viscous fluid, the droplet volume is proportional to the dispersed flow rate. Consequently the size of the PLGA droplets is larger in a higher oil flow rate. The effect of the oil/water flow rates on the trend of the droplet size corresponds well with the prediction of previous literatures [36–38]. It is worth noting that the small relative standard deviation (RSD, defined as the ratio of the standard deviation to the average) in droplet size in Table 1 reflects the good uniformity of the PLGA droplets obtained under each flow condition. This RSD agrees with the results of the monodispersed images in Figure 5.

This study successfully applied an aluminum microfluidic chip fabricated by mechanical micromachining to generate uniform PLGA microparticles which were made by our previous polydimethylsiloxane (PDMS)-based chip [39]. The proposed aluminum chip has the following advantages over the chip in our previous report: (i) the aluminum substrate has a high chemical resistance to organic solvents. Based on our experience, PDMS chips generally have a low chemical resistance to some organic solvents (such as dichloromethane in this study). When dichloromethane is used, a PDMS chip may swell, degrade, and/or deform over time; (ii) a rigid aluminum chip has good mechanical properties, is durable and wears well; (iii) The aluminum chip is easily disassembled for cleaning and reuse than the thermal bonded PDMS chip.

However, the present technology of mechanical micromachining cannot easily perform miniaturization work smaller than 50 micrometers in channel size [5,16]. This method is also limited in the size of small microparticles it can produce due to the size limit of creating microchannels. However, the applications of the proposed method show their potential for use as micro-carriers for biological labeling and diagnostics, in micro-mixers, micro-reactors (synthesis of metal nanoparticles, such as gold, silver, platinum, magnetic ion oxide, and alloy particles), and the generation of various polymer beads (micro-scaled polymers could be used for oral usage, as well as being a vehicle for drug/enzyme controlled release).

## 4. Conclusions

This study demonstrated a simple and impressively robust aluminum-based microfluidic chip for fabricating size-controlled and monodispersed CdSe/ZnS-TOPO QDs-loaded PLGA microparticles. From a practical point of view, aluminum is a suitable substrate for a microfluidic chip due to its excellent mechanical properties, and the fact that it is readily fabricated by mechanical micromachining. Based on the outstanding performance of the microfluidic technique, we utilized the flow-focusing design to generate extremely ordered and regular microparticles. By adjusting the water/oil flow rates, we were able to manufacture microparticles of precisely controlled and monodispersed size distributions. Based on the results of this study, our proposed Al-based microfluidic chip can be used for the preparation of various monodispersed microparticles, especially for encapsulating biological entities for labeling pharmaceuticals

## Supplementary Information



## Figures and Tables

**Table 1.** The relationships between particle size and flow rates of the two phases.

**Flow rate of water phase (mL/min)**	**Flow rate of oil phase (mL/min)**	**Droplet**		**Microparticle**		**Shrinkage (%)**
		**Size (μm)**	**RSD (%)**	**Size (μm)**	**RSD (%)**	
0.08	0.06	382.1	1.6	35.1	5.8	90.8
	0.065	402.9	2.1	35.8	10	91.1
	0.07	481.3	1.9	41.1	8.1	91.5
	0.075	579	1.5	48.2	7.7	91.7
	0.08	654.9	1.2	58.2	8	91.1
0.10	0.06	318.9	1.9	27.8	5.9	91.3
	0.065	376.9	1.4	34.1	6	91
	0.07	432.5	1.4	38.5	2.9	91.1
	0.075	510.5	1.2	42.6	5.4	91.7
	0.08	520	1.2	42.8	5.3	91.8
0.12	0.06	301.6	2	25.9	8.8	91.4
	0.065	321.5	2	30.2	6.8	90.6
	0.07	338.8	1.8	30.6	6.3	91
	0.075	351.1	2.5	31.2	9.7	91.1
	0.08	372.9	1.4	31.6	7.8	91.5
