# Supplementary material for: An Aluminum Microfluidic Chip Fabrication Using a Convenient Micromilling Process for Fluorescent Poly(dl-lactide-co-glycolide) Microparticle Generation
Source: Sensors (Basel). 2012 Feb 1;12(2):1455–67. doi: 10.3390/s120201455 (PMC3304121; doi:10.3390/s120201455)
Supplement: Supplementary file 1 [file sensors-12-01455-s001.pdf]

Supplementary Information

Yung-Sheng Lin [1,2], Chih-Hui Yang [1], Chih-Yu Wang [3], Fang-Rong Chang [4], Keng-Shiang Huang [5,*] and Wan-Chen Hsieh [1]

1 Department of Biological Science & Technology, I-Shou University, Kaohsiung 84001, Taiwan; E-Mails: linys@sunrise.hk.edu.tw (Y.-S.L.); chyang@isu.edu.tw (C.-H.Y.); wanjenxie@gmail.com (W.-C.H.)

2 Department of Applied Cosmetology and Master Program of Cosmetic Science, Hung-Kuang University, Taichung 43302, Taiwan

3 Department of Biomedical Engineering, I-Shou University, Kaohsiung 82445, Taiwan; E-Mail: crab@isu.edu.tw

4 Graduate Institute of Natural Products, College of Pharmacy, Kaohsiung Medical University, Kaohsiung 807, Taiwan; E-Mail: aaronfrc@kmu.edu.tw

5 The School of Chinese Medicine for Post-Baccalaureate, I-Shou University, Kaohsiung 82445, Taiwan

* Author to whom correspondence should be addressed; E-Mail: huangks@isu.edu.tw.

Received: 30 December 2011; in revised form: 19 January 2012 / Accepted: 31 January 2012 / Published: 1 February 2012

S1. Preparation of TOPO-Coated CdSe/ZnS QDs

The synthesis of tri-n-octylphosphine oxide (TOPO)-coated CdSe/ZnS QDs was modified from the method proposed in previous literatures [1,2]. In brief, 0.3 g of CdO, 0.65 g of n-tetradencylphosphonic acid and 25 g of TOPO were loaded into a 100 mL flask, and the mixture was then heated to 350 °C under an argon flow. When the temperature of the solution was cooled down to 250 °C, a Se stock solution prepared from 0.592 g of Se powder dissolved in 15 mL of tributylphosphine was injected into it and the QDs grew at 250 °C for 90 s. Then, 2 mL of ZnS precursor solution, which is a mixture of 10 mL of 2.0 M $Zn(CH_3)_2$ in toluene and $S(Si(CH_3)_3)_2$ in TBP, was added drop-wise to this mixture. The heater was then stopped and the crude TOPO-coated CdSe/ZnS was synthesized. After drying under vacuum, 0.22 g of TOPO-coated CdSe/ZnS was obtained. CdSe has a band gap of 1.7 eV [3] and the emission colour of the nanoparticles shifted continuously from red (centered at 650 nm) to blue (centered at 450 nm) as the size of the nanoparticles decreased. The emission colour of the prepared CdSe/ZnS in this work was green (554 nm), which is similar to the result of previous studies [1].

S2. Characterization

Characterization of CdSe/ZnS-TOPO QDs-loaded PLGA microparticles was carried out by an optical microscope and a scanning electron microscope (SEM, S-4300, Hitachi, Japan). The optical detection system consisted of an optical microscope (TE2000U, Nikon, USA) and a digital camera (Evolution Color VF, Nikon, USA). The recorded pictures were analyzed by the homemade images

analysis software in MATLAB (MathWorks Inc., USA) on a Pentium4-3.4G computer to determine the average size of the PLGA microparticles. To ensure a statistically representative size, more than 100 particles were counted.

S3. Influence of Organic Solvents

Common PDMS chips will deform when they come in contact with dichloromethane (Figure S2). Conventional glass chips are difficult to clean because of the bonded fixation design [4–6]. The proposed Al microfluidic chips have the advantages of being durable, with a high level of chemical resistance to organic solvents, and are designed to easily disassemble for channel rinsing and cleaning.

Figure S1. Transmission electron microscope (TEM) micrographs of TOPO-coated CdSe/ZnS QDs deposited from dispersions in chloroform.

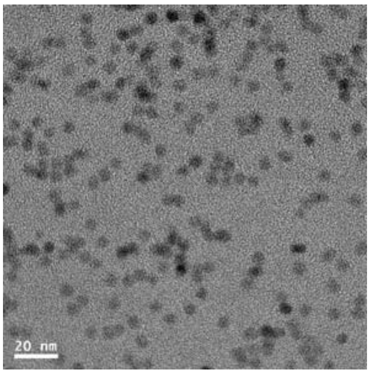

Figure S2. The PDMS chip before (**A**) and after (**B**) contacting with dichloromethane.

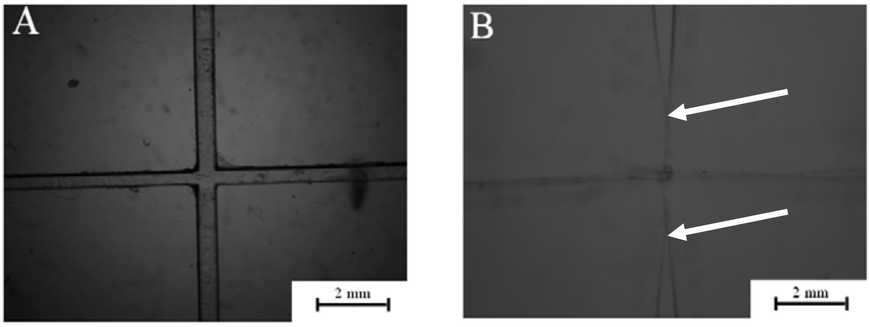

Figure S3. Size distribution of PLGA droplets as shown in Figure 5.

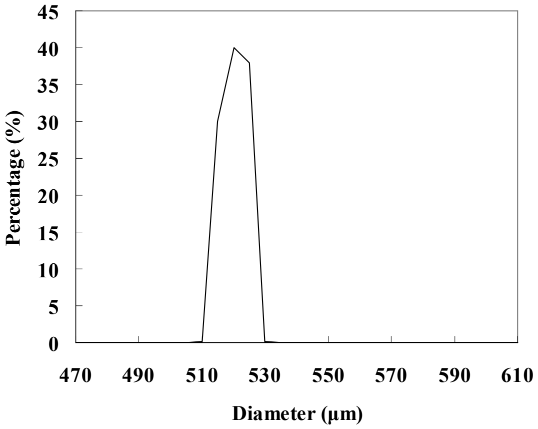

References

1. Hines, M.A.; Guyot-Sionnest, P. Synthesis and characterization of strongly luminescing ZnS-capped CdSe nanocrystals. *J. Phys. Chem.* **1996**, *100*, 468–471.
2. Peng, Z.A.; Peng, X. Formation of high-quality CdTe, CdSe, and CdS nanocrystals using CdO as precursor. *J. Am. Chem. Soc.* **2001**, *123*, 183–184.
3. Kortan, A.R.; Hull, R.; Opila, R.L.; Bawendi, M.G.; Steigerwald, M.L.; Carroll, P.J.; Brus, L. Nucleation and growth of CdSe on ZnS quantum crystallite seeds, and vice versa, in inverse micelle media. *J. Am. Chem. Soc.* **1990**, *112*, 1327–1332.
4. Lee, M.; Lee, J.; Rhee, H.; Choo, J.; Chai, Y.G.; Lee, E.K. Applicability of laser-induced Raman microscopy for *in situ* monitoring of imine formation in a glass microfluidic chip. *J. Raman Spectrosc.* **2003**, *34*, 737–742.
5. Jia, Z.J.; Fang, Q.; Fang, Z.L. Bonding of glass microfluidic chips at room temperatures. *Anal. Chem.* **2004**, *76*, 5597–5602.
6. Qu, B.Y.; Wu, Z.Y.; Fang, F.; Bai, Z.M.; Yang, D.Z.; Xu, S.K. A glass microfluidic chip for continuous blood cell sorting by a magnetic gradient without labeling. *Anal. Bioanal. Chem.* **2008**, *392*, 1317–1324.

© 2012 by the authors; licensee MDPI, Basel, Switzerland. This article is an open access article distributed under the terms and conditions of the Creative Commons Attribution license (http://creativecommons.org/licenses/by/3.0/).
